# Latent tuberculosis in nursing professionals of a Brazilian hospital

**DOI:** 10.1186/1745-6673-6-15

**Published:** 2011-05-17

**Authors:** Karen Gisele Person Severo, Julia da Silva Oliveira, Marcelo Carneiro, Andréia Rosane de Moura Valim, Eliane Carlosso Krummenauer, Lia Gonçalves Possuelo

**Affiliations:** 1 Acadêmica do Curso de Farmácia.Universidade de Santa Cruz do Sul. Avenida Independência, 2293- Bloco 35- Bairro Universitário. 96815-900 - Santa Cruz do Sul, RS. Caixa-Postal: 188, Brasil; 2 Comissão de Controle de Infecção Hospitalar, Hospital Santa Cruz. Rua Fernando Abott, 174- Bairro Centro. 96810-072 - Santa Cruz do Sul, RS, Brasil; 3 Departamento de Biologia e Farmácia - Universidade de Santa Cruz do Sul Universidade de Santa Cruz do Sul. Avenida Independência, 2293-Bloco 12- Bairro Universitário. 96815-900 - Santa Cruz do Sul, RS. Caixa-Postal: 188, Brasil; 4 Laboratório de Genética e Biotecnologia, Universidade de Santa Cruz do Sul.Universidade de Santa Cruz do Sul. Avenida Independência, 2293-Bloco 20- Bairro Universitário. 96815-900 - Santa Cruz do Sul, RS. Caixa-Postal: 188, Brasil

**Keywords:** Tuberculosis, Tuberculin Skin Test, Health-Care Workers

## Abstract

Tuberculosis (TB) is considered an occupational disease among health-care workers (HCWs). Direct contact with TB patients leads to an increased risk to become latently infected by *Mycobacterium tuberculosis*. The objective of this study is to estimate the prevalence of latent *M. tuberculosis *minfection among nursing professionals of a hospital in Rio Grande do Sul, Brazil, assessed by tuberculin skin test (TST). From November 2009 to May 2010, latent *M. tuberculosis *infection was assessed by TST in 55 nursing professionals. Epidemiological information was collected using a standardized questionnaire. A positive TST result (> or = 10 mm) was observed in 47.3% of the HCWs tested. There was no significant difference in TST positivity when duration of employment or professional category (technician or nurse) was evaluated. The results of this work reinforce the need for control measures to prevent latent *M. tuberculosis *infection among nursing professionals at the hospital where the study was conducted.

## Introduction

Tuberculosis (TB), mainly caused by *Mycobacterium tuberculosis*, is one of the most ancient and neglected diseases of humanity[1]. According to the World Health Organization (WHO), one third of the world's population, around 1.7 billion people, are infected with TB [2].

Health-care workers (HCWs) present a higher risk of infection compared to the general population. A longer period of employment as health professional, patient's delayed diagnosis of the disease, professional category, certain work locations such as inpatient TB facility, laboratory, internal medicine, and emergency facilities, in addition to the lack of proper respiratory protection (N95 masks), are factors that can contribute to the infection [3-5].

The adoption of measures to control the transmission of the disease in the work environment can be helpful in decreasing the incidence of the disease in the population. Declaring that a disease is an occupational one is an important warning, so that specific control measures can be taken in order to avoid its dissemination among institution employees [4,6].

The risk of infection will depend on many factors such as: amount of bacilli expelled by the patient; duration of patient's infectiousness; bacillus concentration in the air, determined by ventilation; exposure time and individual susceptibility. There are no safe levels of exposure to TB. Currently, the Center for Disease Control (CDC) and the Occupational Safety and Health Administration (OSHA) acknowledge that in centers where appropriate control measures are applied the risk of contracting TB among HCWs is comparable to that in the community where they live. TST testing is still a low-cost strategy for the screening of health-care workers infected by *M. tuberculosis*.

There are few data about the prevalence of *M. tuberculosis *infection among HCWs in south Brazil, hence, the aim of the present study is to estimate the prevalence of latent TB infection (LTBI) and to evaluate the characteristics related to it among HCWs from Santa Cruz Hospital, in the state of Rio Grande do Sul, Brazil.

## Methods

A descriptive study was conducted from November 2009 to May 2010. The subjects included in the study were nurses and nurse technicians who had been employed for longer than three months, worked at least 6 hours in São Francisco and Santo Antônio wards and adult Intensive Care Unit (ICU) of Hospital Santa Cruz. Santa Cruz hospital is a teaching hospital of middle complexity. Those three wards were selected because hospitalized TB patients are treated there.

HCWs enrolled in the study signed an informed consent and filled in a standardized questionnaire containing question about sociodemographic features, BCG vaccination history, duration of employment, possible exposure to TB in their households and in the hospital, HIV infection and corticosteroid use. Vaccination was assessed by the presence of a BCG scar in the right arm. Following the national vaccination plan, BCG vaccination for newborns is mandatory in Brazil and, until 2006 was repeated in children aged 6-10 years. HCWs who did not return for TST reading within the recommended timeframe were excluded from the study. The TST was performed by a trained professional after a brief explanation of possible results of the test. The Mantoux technique was used and 0.1 mL of Purified Protein Derivative (PPD-Rt 23) was applied by an intradermal injection in the middle third of the inner forearm of HCWs. The induration was measured by a single trained professional, 48 to 72 hours after the injection. A positive TST was defined as an induration ≥10 mm.

This project was approved by the Research Ethics Committee of Universidade de Santa Cruz do Sul - UNISC protocol nº 2421/09 and was not granted financial support. Clinical data were entered in a SPSS 12.0 software database for statistical analysis. Fisher's exact test and χ^2 ^test were used, and a *p *value of <0.05 was considered significant.

## Results

A total of 65 HCWs are allocated at the selected wards (12 nurses and 53 nurse technicians). They were all tested but TST results were available for 55 individuals (84.6%).

Among the participant HCWs, 48 (87.3%) were female, 53 (96.4%) were self-declared Caucasians and mean age was 29.9 ± 6.7 years. Forty-four (80%) were nurse technicians and 11 (20%) were nurses. As for duration of employment, 13 (23.6%) had been in the workplace for less than one year. The BCG scar was observed in 54 (98.2%) HCWs and 6 (10.9%) were smokers. None of the individuals was undergoing corticosteroid treatment (Table 1).

**Table 1:** Univariate analysis of characteristics related to TST results in HCWs.

Variables	TST Positive N = 26 (%)	TST Negative N = 29 (%)	*Total* N = 55 (%)	*P*
**Gender**				
Male	4 (57.1)	3 (42.9)	7 (12.7)	0.69
Female	22 (45.8)	26 (54.2)	48 (87.3)	
**Skin color**				
Caucasian	25 (47.2)	28 (52.8)	53 (96.4)	1.00
Non-Caucasian	1 (50)	1 (50)	2 (3.6)	
**Age**				
<30 years	13 (43.3)	17 (56.7)	30 (54.5)	0.71
≥30 years	13 (57.1)	12 (42.9)	25 (45.5)	
**Occupation**				
Nurse technician	20 (45.5)	24 (54.5)	44 (80.0)	0.41
Nurse	6 (54.5)	5 (45.5)	11 (20.0)	
**BCG Scar**				
Yes	26 (48.1)	28 (51.9)	54 (98.2)	-
No	0	1 (100)	1 (1.8)	
**Smoker**				
Yes	1 (16.7)	5 (83.3)	6 (10.9)	0.19
No	25 (51.0)	24 (49)	49 (89.1)	
**Duration of employment**				
<1 year	7 (63.6)	4 (36.4)	11 (20)	0.19
≥ 1 year	19 (43.2)	25 (56.8)	44 (80)	
**Workplace**				
São Francisco Ward	12 (54.5)	10 (45.5)	22 (40.0)	0.46
Santo Antônio Ward	8 (53.3)	7 (46.7)	15 (27.3)	
Adult ICU	6 (33.3)	12 (66.7)	18 (3.7)	

All 55 HCWs reported having had contact with TB-infected patients during their professional activities. A positive TST was observed in 26 (47.3%) individuals, regardless of the occupation (technician or nurse) (*p *= 0.41) or the ward they worked at (*p *= 0.46) (Table 1). As for employment duration, HCWs who had been in the job for less than one year presented a higher positivity rate in comparison to those working for longer than one year (*p *= 0.06).

## Discussion

TST, described in the 19th century, remains a good tool for the diagnosis of *M. tuberculosis *infection. It is indicated for people who are at risk of infection and progression to active disease, people who would benefit from prophylactic treatment with isoniazid. Therefore, tuberculin reactiveness surveys have been recommended for HCWs, immunosuppressed individuals and those who have had contact with TB patients with active disease [7].

In this study, 47.3% of TST positivity was observed among HCWs from Hospital Santa Cruz. Similar studies involving HCWs described high TST positivity rates, ranging from 26.7% to 69.5% [8-11]. Muzy de Souza (2000) reported 51% of TST positivity in HCWs in a general hospital in Rio de Janeiro, similar to the data of our study [10]. The high prevalence of LTBI in this population of professionals is strongly associated to the presence of TB patients (diagnosed or not) at the workplace, which reflects the epidemiological situation of the disease in the community [11]. In a study conducted by Demkow *et al*. (2008), HCWs that assisted patients with active TB, showed a prevalence of 27.1% of *M. tuberculosis *infection and risk of having a positive TST was associated to certain activities at work. The laboratory technicians presented a positivity rate as high as 50%, while physicians from the TB department and nurses had 34% and 30%, respectively, and administrative personnel, 15% [12]. In our study, there was no significant difference in TST positivity between the professional categories analyzed (*p *= 0,41). Perhaps a larger sample size might have resulted in the detection of a significant difference. The high proportion of LTBI in HCWs evaluated could also be related to the effect of BCG vaccination. The influence of BCG vaccination on TST was not investigated in the present study because non-vaccinated HCWs were not available. In the future studies, another alternative method to TST, such as interferon-gamma release assays (IGRA), could be carried out to exclude possible false-positive due to BCG vaccination. Torres Costa (2009) reported in a study performed among HCWs in a Portuguese hospital that the effect of BCG vaccination or repeated BCG vaccination could be related with the probability of positive TSTs. The TST was used in the present study because the IGRA was not available in our laboratory at the time of this study. Future studies could be performed to compare the sensitivity and specificity between TST and IGRA in the studied population.

From 1999 to 2000, a study including 4,419 HCWs employed at four hospitals in three different Brazilian states was carried out, in which TST was positive in 63% of individuals and conversion rate was 9% [14]. Risk factors associated to TST conversion were nosocomial exposure to pulmonary TB patients, professional category and the lack of implemented biosafety measures [14]. Implementation of consecutive administrative and personal infection-control measures in addition to educational measures can significantly reduce the risk of latent TB infection [4].

Some studies reported the occurrence of a booster effect ranging from 5.8% to 7.8% [5,15]. The booster effect was not evaluated in our study. Conde *et al*. (2009) reported in the III Brazilian Consensus for Tuberculosis that the booster effect testing is not needed when HCWs are evaluated [7].

Many risk factors for TB bacillus infection have been reported [7,16]. The most common are overcrowding, alcohol abuse, age, gender, skin color and corticosteroid treatment. In the present study, some of these characteristics were evaluated and no significant association to TST was observed.

In our study, we observed a higher frequency of TST positivity among HCWs who had been at the job for less than one year (63.6%). This could represent a previously acquired infection while working as a healthcare professional or a recently acquired infection during their work in the hospital. However, we cannot be certain of that, as TST is not routinely applied as a pre-employment screening test. In a study conducted among HCWs in a school hospital, the risk for a positive TST increased after one year at the job, suggesting that prevention measures should be applied to those people initiating health profession careers [4,10].

The high prevalence of a positive TST among HCWs located at São Francisco (55%) and Santo Antônio (53.3%) wards was already expected because those individuals directly assist TB patients in the initial phase of treatment or patients without a confirmed diagnosis; thus, respiratory protection measures are not always taken.

In developing countries, the risk of infection/disease among HCWs could be drastically reduced if governmental and health authorities actually considered TB control a priority. HCWs are a valuable resource for infection control, as long as they comply with it, for instance, taking personal self-protective measures such as raising TB awareness in patients hospitalized due to a pneumopathy. An educational program is primordial for the understanding of basic concepts of disease transmission and symptoms, in addition to the importance of personal protective measures for TB control [17].

Despite the small sample size, we observed a high prevalence of TST positivity (47.3%) among the HCWs analyzed. Other similar studies should be conducted in different hospitals and healthcare facilities in order to complement these data and emphasize the need to implement biosafety guidelines and the importance of the correct use of personal protective equipment by HCWs. The results of our study provide data for hospital infection control committees, safety engineering specialized services and occupational medicine to develop preventive measures to reduce the LTBI rates among their employees.

## Competing interests

The authors declare that they have no competing interests.

## Authors' contributions

KS: Conception, design, data collection, analysis and interpretation and drafting of the manuscript. JSO: Conception, design and involved in drafting of the manuscript. MC: Conception, design and critical review.

ARMV: Conception, design and critical review. EK: Conception and design and involved in the drafting of the manuscript. LGP: Conception and design, data analysis and interpretation.

All authors read and approved the final manuscript.
